# A case of mantle cell lymphoma presenting as IgG4-related dacryoadenitis and sialoadenitis, so-called Mikulicz’s disease

**DOI:** 10.1186/s12957-015-0644-0

**Published:** 2015-07-25

**Authors:** Yoshikazu Hayashi, Masafumi Moriyama, Takashi Maehara, Yuichi Goto, Shintaro Kawano, Miho Ohta, Akihiko Tanaka, Sachiko Furukawa, Jun-Nosuke Hayashida, Tamotsu Kiyoshima, Mayumi Shimizu, Toru Chikui, Seiji Nakamura

**Affiliations:** Section of Oral and Maxillofacial Oncology, Division of Maxillofacial Diagnostic and Surgical Sciences, Faculty of Dental Science, Kyushu University, 3-1-1 Maidashi, Higashi-ku, Fukuoka, 812-8582 Japan; Laboratory of Oral Pathology, Division of Maxillofacial Diagnostic and Surgical Sciences, Faculty of Dental Science, Kyushu University, 3-1-1 Maidashi, Higashi-ku, Fukuoka, 812-8582 Japan; Department of Oral and Maxillofacial Radiology, Kyushu University Hospital, 3-1-1 Maidashi, Higashi-ku, Fukuoka, 812-8582 Japan

**Keywords:** Mantle cell lymphoma, IgG4-related dacryoadenitis and sialoadenitis, Lacrimal glands, Salivary glands

## Abstract

**Background:**

Mantle cell lymphoma (MCL) is a relatively uncommon type of non-Hodgkin lymphoma. It develops in the outer edge of a lymph node called the mantle zone. In contrast, IgG4-related dacryoadenitis and sialoadenitis (IgG4-DS) is characterized by elevated serum IgG4 and persistent bilateral enlargement of lacrimal glands (LGs) and salivary glands (SGs), with infiltration of IgG4-positive plasma cells. Recent studies indicated the importance of differentiation between IgG4-DS and malignant lymphoma.

**Case presentation:**

An 82-year-old man was suspected of IgG4-DS because of a high serum IgG level (2174 mg/dL) and bilateral swelling of LGs and SGs. Lip biopsy and fine needle biopsy of submandibular gland were performed, and subsequently, MCL was diagnosed through the histopathological findings.

**Conclusions:**

MCL most commonly occurs in the Waldeyer ring, but rarely in the stomach, spleen, skin, LG, and SG. We report an unusual case of MCL involving LGs and SGs mimicking IgG4-DS, which suggests that IgG4 testing may be useful in the differentiation of IgG4-DS in the presence of bilateral swelling of LGs or SGs.

## Background

Mantle cell lymphoma (MCL) is a relatively rare type of non-Hodgkin lymphoma, accounting for ~3 % of malignant lymphoma in Japan. MCL typically occurs in middle-aged to older adults, with a marked male predilection, and has a poorer prognosis compared with other subtypes of non-Hodgkin lymphoma [1, 2]. MCL more often presents in stage III/IV with lymphadenopathy, hepatosplenomegaly, bone marrow involvement, and leukemic spread [3]. Although the majority of MCLs occur in lymph nodes, 25 % of patients have extranodal involvement, with the Waldeyer ring (6.3 %), intestine (5 %), stomach (2.5 %), orbit (2.5 %), and salivary gland (2.5 %) [4]. Definitive diagnosis of MCL is predicated on appropriate immunohistochemical staining with or without ancillary molecular and flow cytometric studies. Clinicopathological characteristics of MCL are characterized by overexpression of cyclin D1 protein; a feature not seen in other similar-appearing lymphomas.

IgG4-related dacryoadenitis and sialoadenitis (IgG4-DS), also known as Mikulicz’s disease, is a unique condition characterized by enlargement of the lacrimal glands (LGs) and salivary glands (SGs) caused by infiltration of lymphocytes. IgG4-DS has been considered to be a subtype of Sjögren’s syndrome (SS) because of certain histopathological similarities, particularly lymphocytic infiltration [5]. However, IgG4-DS patients show elevated serum levels of IgG4 and infiltrating IgG4-positive plasma cells in the glandular tissues [6, 7]. Similar findings have also been identified in other diseases such as autoimmune pancreatitis [8], interstitial pneumonia [9], retroperitoneal fibrosis [10], and sclerosing cholangitis [11], and these diseases are now referred to as IgG4-related disease (IgG4-RD) [12]. IgG4-DS is now diagnosed by both “Comprehensive Diagnostic Criteria for IgG4-related Disease (2011)” and “Diagnostic Criteria for IgG4-related Mikulicz’s Disease” approved by the Japanese Society for Sjögren’s syndrome [13]. However, it is important to differentiate IgG4-RD from malignant tumors (such as cancer and lymphoma) and similar diseases (such as SS, primary sclerosing cholangitis, Castleman’s disease, secondary retroperitoneal fibrosis Wegener’s granulomatosis, sarcoidosis, and Churg–Strauss syndrome) by histopathological examination of local lesions. Recently, we reported a case of marginal zone B-cell lymphoma mimicking IgG4-DS [14]. Here, we report a rare case of MCL involving multiple lymph nodes and bilateral LGs and SGs mimicking IgG4-DS.

## Case presentation

An 82-year-old man was referred to our institution with bilateral swelling of the LGs and submandibular glands (SMGs) in November 2008. He had previously attended the ophthalmology department in our hospital with chief complaints of bilateral swelling of LGs, which were diagnosed as IgG4-DS by these clinical findings and high serum IgG (2174 mg/dL). LG biopsy was performed for definitive diagnosis and 2 days later he visited our institution on referral from our ophthalmology department for further evaluation of SMGs. He had several medical histories of colonic cancer, hypertension, and cerebral infarction.

**Fig. 1:**
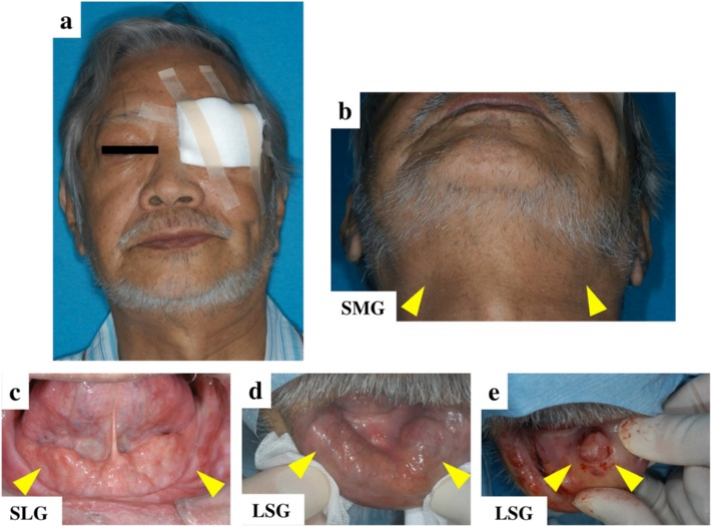
Clinical findings before treatment. Bilateral swelling of LGs, SMGs, and SLGs (*yellow arrowheads*) (**a**–**c**). Multiple swelling of LSGs at the time of biopsy (*blue arrowheads*) (**d**, **e**)

**Fig. 2:**
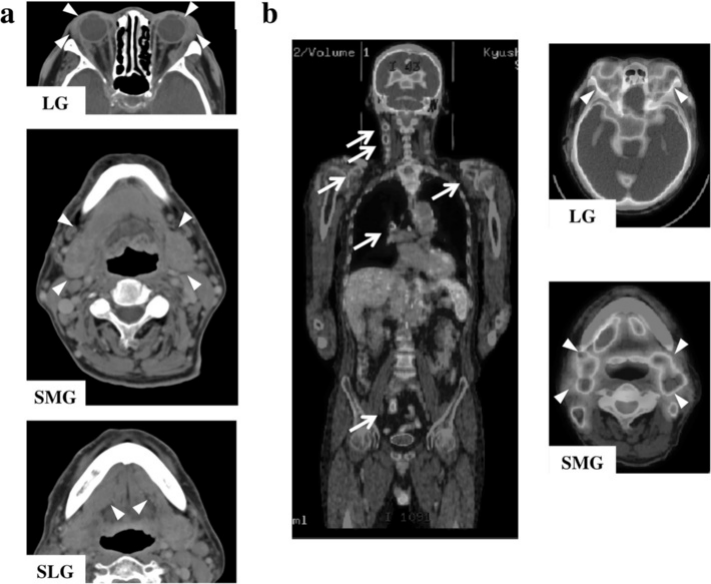
Imaging findings before treatment. **a** CT findings indicating swelling of LGs, SMGs and SLGs (*white arrowheads*). **b** FDG-PET indicating abnormal multiple accumulations in SMGs and LGs (*white arrowheads*) and in multiple systemic lymph nodes (*white arrows*)

Physical findings showed no fever (body temperature, 36.3 °C), blood pressure of 132/78 mmHg, no weight loss, and no nocturnal sweating. He was aware of dry mouth (unstimulated salivary flow rate, 0.4 mL/15 min). Bilateral SMGs, sublingual glands (SLGs), and labial salivary glands (LSGs) were elastic, hard and swollen without pain (Fig. 1). Several cervical lymph nodes were enlarged. Computed tomography (CT) showed marked swelling of bilateral LGs and SMGs (Fig. 2a). F-18 fluorodeoxyglucose positron emission tomography demonstrated abnormal multiple accumulations in the LGs (SUVmax, 7.14), SMGs (SUVmax, 6.83), and several systemic lymph nodes (SUVmax, 3.45–4.74), including in the neck and mediastinum (Fig. 2b).

He had a low hemoglobin level (13.4 g/dL), red blood cell count of 4.57 × 10^6^/mm^3^, white blood cell count of 7150/mm^3^ (neutrophils 45.5 %, lymphocytes 35.0 %, monocytes 9.5 %, eosinophils 1.0 %, and basophils 0.5 %), and platelet count of 179 × 10^3^/mm^3^. His C-reactive protein level was 0.23 mg/dL. The serum soluble interluekin-2 receptor concentration was abnormally high (2251.4 U/mL). Immunological tests were negative for the anti-SS-A/B antibody and the anti-thyroid peroxidase antibody, but positive for rheumatoid factor. Serum levels of IgA, IgM, and IgE were within normal limits (354 mg/dL, 167 mg/dL, and 44 IU/mL, respectively), but his serum IgG level was elevated (2174 mg/dL).

**Fig. 3:**
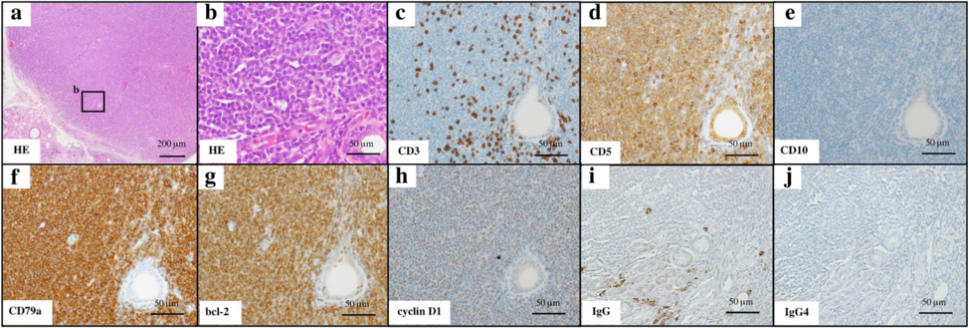
Histological findings in LSG. Marked lymphoplasmacytic infiltration with hyperplastic lymphoid follicles. The infiltrating lymphocytes were stained with hematoxylin and eosin (HE) (**a**, **b**), anti-CD3 (**c**), anti-CD5 (**d**), anti-CD10 (**e**), anti-CD79a (**f**), anti-bcl-2 (**g**), anti-cyclin D1 (**h**), IgG (**i**), and IgG4 (**j**) monoclonal antibodies

IgG4-DS was suspected because of persistent symmetrical swelling of at least two pairs of LGs and major SGs for at least 3 months. We thus additionally examined serum IgG4 and it was in the normal range (43.7 mg/dL). We performed LSG biopsy and fine needle biopsy (FNB) of the left SMG in addition to LG biospy to check if LSG and SMG were the same disease. Histologically, all sections showed severe uniform infiltration of lymphoplasmacytes, without lymphoid follicular formation. The plasmacytoid cells showed nuclear pleomorphism. Immunohistochemical staining showed monotypic predominance of kappa-light chain and no infiltration of IgG4-positive plasma cells. The infiltrating lymphocytes were positive for B-cell markers (CD20 and CD79a), CD5, bcl-2, and cyclin D1, but negative for T-cell markers (CD3) and CD10 (Fig. 3).

These histopathological findings and clinical features confirmed a diagnosis of MCL. The patient underwent radiotherapy (20 Gy/10 Fr) and chemotherapy (600 mg rituximab, four times). Although swelling of the LGs and SMGs diminished markedly, it became intractable to treatment at 1 year following therapy and finally terminated in patient death in November 2011.

## Conclusions

MCL is recognized as an aggressive lymphoma for which the median survival of patients is ~3 years [15]. The clinical and prognostic implications of initial presentation of MCL in the hard palate are currently unknown. MCL is presently staged by using a modified Ann Arbor system [16]. Stage I consists of the involvement of a lymphoid structure or lymph node region, whereas stage II exhibits the involvement of two or more regions on the same side of the diaphragm. Stage III disease represents the involvement of structures or lymph node regions on both sides of the diaphragm, whereas stage IV disease is characterized by diffuse disseminated involvement of one or more extralymphatic organs, such as bone marrow, liver, or lung. Our patient had stage II disease at presentation, with lesions of the LGs, SGs, and cervical lymph nodes.

Immunophenotypically, the neoplastic lymphocytes usually express CD5, CD20, CD43, and FMC-7, but not CD10 or Bcl-6 [17]. The lymphocytes are typically negative for CD23 expression, but on occasion, they may be weakly positive. All lymphocytic specimens typically express Bcl-1, including the rare specimen that is CD5 negative [18]. An important, but less-recognized fact is that almost all lymphocytes are Bcl-2 positive. Several studies have reported that immunohistochemical detection of cyclin D1 is useful for diagnosis of MCL [19–21]. In this case, we confirmed that the patient was positive for CD-5 and cyclin D1 and negative for CD-10. This suggested that this case was consistent with MCL (Table 1).

**Table 1:** Differential diagnosis of lymphoma in immunostaining

	CD3	CD5	CD10	CD20	CD79a	Cyclin D1	bcl-2	UCHL-1
DLBL	−	−	+/−	+	+	−	+/−	−
FL	−	−	+	+	+	−	+	−
MALT lymphoma	−	−	−	+	+	−	+	−
MCL	−	+	−	+	+	+	+	−
This case	−	+	−	+	+	+	+	−

*DLBL* diffuse large B-cell lymphoma, *FL* follicular lymphoma, *MALT* mucosa-associated lymphoid tissue

Larry et al. [4] reported clinical characteristics of primary MCLs. According to their study, 20 of 80 MCLs involved extranodal sites, while only two involved SGs. To the best of our knowledge, two cases of MCL were reported to involve bilateral LGs and parotid glands (Table 2) [22, 23]. Therefore, the present case report of MCL occurring in bilateral LGs and SMGs is rare, and these clinical findings were similar to those in IgG4-DS. We thus additionally examined serum IgG4 levels and performed both LSG biopsy and FNB of swelling salivary glands.

**Table 2:** MCL involved in LGs and SGs

Case	Sex	Age	Regions		Ann Arbor staging	Author	Year
1	Female	42	LG, PG, SMG	Bilateral	II	Palaniswamy [22]	2009
2	Male	52	LG, PG, SMG	Bilateral	II	Sagar [21]	2011
3	Male	82	LG, PG, SMG	Bilateral	IIE	This case	2015
			SLG, LSG				

*MCL* mantle cell lymphoma, *LG* lacrimal glands, *SG* salivary gland, *PG* parotid gland, *SMG* submandibular gland, *SLG* sublingual gland, *LSG* labial salivary gland

IgG4-DS is now recognized as a new emerging disorder, characterized by high serum IgG4, marked infiltration of IgG4-positive plasma cells, and severe fibrosis with hyperplastic ectopic germinal centers in LGs and SGs. We recently proposed “Comprehensive Diagnostic Criteria for IgG4-RD” [9]. IgG4-RD can be diagnosed using these criteria combined with organ-specific criteria. If a diagnosis of IgG4-DS is probable or possible based on these criteria, it can be confirmed according to the “Diagnostic Criteria for IgG4-related Mikulicz’s Disease” approved by the Japanese Society for Sjögren’s Syndrome in 2008, which include the following items: (i) persistent (>3 months) symmetrical swelling of more than two LGs and major SGs; (ii) raised serum levels of IgG4 (>135 mg/dL); and (iii) infiltration of IgG4-positive plasma cells in the tissue (IgG4-positive plasma cells/IgG-positive plasma cells >0.4) by immunostaining. For a positive diagnosis of IgG4-DS, any two of these three criteria must be fulfilled, including item (i). The present case met criterion (i), and IgG4-DS was therefore suspected. However, biopsy of the local lesion is recommended for differential diagnosis from other disorders, including sarcoidosis, Castleman’s disease, Wegener’s granulomatosis, lymphoma, and cancer. We therefore performed LSG biopsy and FNB of SMGs, resulting in a definitive diagnosis of MCL. These results suggest that biopsy of the swollen lesion is essential for a definitive diagnosis of IgG4-DS. Moreover, we recently reported the importance of the utility of SMG incisional biopsies [24]. In conclusion, we emphasize the importance of performing thorough biopsy and serum IgG4 testing when making an accurate diagnosis of bilateral LG and SG swelling. Therefore, we suggest that accurate and rapid diagnosis leads to effective treatment.

## Consent

This study design was approved by the Ethics Committee of Kyushu University, Japan, and written informed consent was obtained from all of the patients and healthy controls (IRB serial number: 25–287).

## Abbreviations

CT: computed tomography
IgG4-DS: IgG4-related dacryoadenitis and sialoadenitis
IgG4-RD: IgG4-related disease
LG: lacrimal gland
LSG: labial salivary gland
MCL: mantle cell lymphoma
MD: Mikulicz’s disease
SLG: sublingual gland
SMG: submandibular gland
